# Matt Polyurethane Coating: Correlation of Surface Roughness on Measurement Length and Gloss

**DOI:** 10.3390/polym12020326

**Published:** 2020-02-04

**Authors:** Qiwen Yong, Jinming Chang, Qi Liu, Feng Jiang, Daidong Wei, Haijun Li

**Affiliations:** 1 Institute of Applied Chemistry, College of Chemistry and Chemical Engineering, China West Normal University, Nanchong 637009, China; jinmingchang@cwnu.edu.cn (J.C.); liuqi@cwnu.edu.cn (Q.L.); jiangfeng@cwnu.edu.cn (F.J.); 2 Chemical Synthesis and Pollution Control Key Laboratory of Sichuan Province, China West Normal University, Nanchong 637009, China; 3 Guangzhou Chemical Grouting Co., Ltd., Chinese Academy of Sciences, Guangzhou 510650, China; 4 Key Laboratory of Green Chemistry of Sichuan Institutes of Higher Education, Sichuan University of Science & Engineering, Zigong 643000, China; lihaijun@163.com

**Keywords:** matt polyurethane, AFM, roughness parameter, gloss, surface profilometer

## Abstract

Matt polyurethane coating was successfully prepared through the synergistic effect of castor oil and phenolic epoxy resin into polyurethane backbone. The formation mechanism may be ascribed to the modulus mismatch between the partially modified epoxy polyurethane and partially unmodified polyurethane. Scanning electron microscopy (SEM) was used to observe the micro-rough surface morphologies. Atomic force microscopy (AFM) and three-dimensional (3D) surface profilometer were applied to calculate a series of surface roughness parameters in different dimensions, such as *S_a_*, *S_q_*, *S_p_*, *S_v_*, *S_z_*, *S_ku_*, *S_sk_*, etc. The exciting results of this paper—the correlation of surface roughness on measurement length and gloss—are explored in detail. It reveals the extrinsic property of measured roughness with measurement length and provides guidance for what kind of incident angle gloss meters (20°, 60°, and 85°) best describe the gloss of matt polyurethane coating.

## 1. Introduction

Gloss is an optical property that indicates how well a surface reflects light in a specular (mirror-like) direction. It is one of the most important indicators used to describe the visual effect of an object [1,2,3,4]. The factors affecting gloss are various, including the angle of incident light and surface topography, etc. [5,6,7]. Gloss is not a single parameter, but a number of surface phenomena that constitute the light-reflecting properties of a surface. The most well-known type of gloss, which gives the perception of a shiny surface, is specular gloss [8]. Other types of gloss contain distinctness-of-image gloss, surface-uniformity gloss, and contrast gloss [9,10,11]. This research deals only with specular gloss, which is shorted for gloss. This gloss has been extensively used in the coating industry to describe the reflectance properties of a coating. The specular gloss is defined as a measure of the specular reflectance of a surface relative to the specular intensity reflected by a standard template at an angle of incidence *θ*:

$$G=100\times \frac{I\left(\theta \right)}{I_{0}\left(\theta \right)}$$

where *I* is the intensity of specular reflection of the sample; *I*_0_ is the specular reflectance from the standard template; and *θ* could be 20°, 60°, or 85°.

Surface roughness is an intrinsic property of a surface, which is often quantified by the deviations in the direction of the normal vector of a real surface from its ideal form (e.g., average height: *S_a_*, root mean square height: *S_q_*, maximum peak height: *S_p_*, maximum valley height: *S_v_*, maximum height: *S_z_*, kurtosis: *S_ku_*, skewness: *S_sk_*, etc.) [12]. However, measured (effective) roughness is dependent on the available measurement scale and the sampling interval of the measurement technique. This makes measured roughness essentially an extrinsic property. Therefore, the relationship between roughness and measuring length scale is still an unresolved scientific problem.

Specular gloss and surface roughness are important factors of coatings as they influence the visual perception of a coating on products [13]. Especially for matt coatings, these two parameters need more stringent controls [14,15]; scientists and technologists expect matt films to have a very low-gloss surface as well as a micro-scale rough surface, which can impart good transparency and pleasant touch properties. As a result, many of techniques, such as an additional matting agent [16,17] and photopolymerization [18,19], have been widely used to construct rough surface of a coating. However, they are different from the rough surface naturally formed by the film and are limited by the disadvantages of requiring a complicated physical process or a specially designed equipment and formulation. Thus, there is urgency in developing a matt coating that can produce low-gloss effect by resin itself.

In this research, we demonstrated a simple method to develop a micro-rough surface of a coating by buckling and wrinkling formation as a consequence of mechanical instability produced upon cross-linking of polyurethane with an epoxy resin. By taking advantage of this synthesized matt polyurethane coating, we here investigated the gloss at different incident angles (20°, 60°, and 85°), which are better able to show the matt characteristics of this polyurethane coating. We also examined the surface roughness using atomic force microscopy (AFM) and three-dimensional (3D) surface profilometer at a wide range of measurement length scales. Meanwhile, the correlation of the measurement length scale and surface roughness parameters was revealed. This is the first time to disclose the extrinsic property of surface roughness with measured length scale. Furthermore, we established the relationship between the gloss and surface roughness. Taking their mutual relationships into account can provide guidance for what kind of incident angle gloss meters (20°, 60°, and 85°) are best suited to describe the specular gloss of matt polyurethane coating.

## 2. Light Scattering by Microroughness

In this section, the light scattering phenomena on a randomly micro-rough surface will be presented and discussed. This allows us to understand the specular gloss and the surface roughness more clearly, as well as their relationships based on optic theory. The solution of the scalar wave equation under the Kirchhoff approximation [20] is a useful and widely used model for light scattering from a randomly micro rough surface. It is assumed that the light scattering from a micro-rough surface consists of a specular spike and a directional-diffuse lobe.

$$I\left(\theta \right)/I_{0}\left(\theta \right)=exp-{\left(4\pi \sigma \;cos\;\theta /\lambda \right)}^{2}+D\left(\theta \right)$$

Here, *θ* and *λ* are the wavelength and angle of incident light, respectively, and *σ* is equal to the value of surface roughness parameter *S_q_*. The directional-diffuse components *D*(*θ*) depend on the roughness amplitude, correlation length, and measurement geometry. For a surface with root mean square roughness amplitude *σ*, the specular intensity can be expressed by [21]

$$I\left(\theta \right)/I_{0}\left(\theta \right)=exp-{\left(4\pi \sigma \;cos\;\theta /\lambda \right)}^{2}$$

This result is very well known and has been widely used to estimate root mean square roughness *σ* from measurements of specular intensity as a function of wavelength and incident angle.

**Figure 1:**
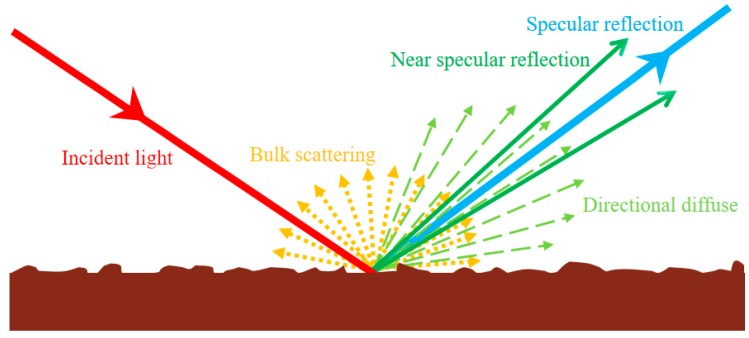
A systematic illustration of light scattering at a randomly micro rough surface.

Nevertheless, the real light scattering from a rough, particulate surface would be even more complicated. For example, coatings, especially containing pigment and filler, exhibit strong bulk scattering. Some light refracted into the body of the film undergoes multiple scattering and a fraction re-emerges from the surface, often with a Lambertian distribution [22]. Relatively large but infrequent surface irregularities, such as bubbles and particle aggregates, may lead to some specular reflection at angles rather than the nominal specular angle, which is often called near specular reflection. A schematic of light scattering behavior of a randomly micro-rough surface can be seen in Figure 1. In general, although there is no single equation that can cover all the light scattering behavior, it seems that the Kirchhoff scalar approximation offers an available model to deal with many real-world surfaces, like coatings and coated paper. There is a limitation which the curvature radius of the micro-roughness features should be larger than the wavelength of incident light.

## 3. Experimental

### 3.1. Materials

Castor oil (CO; chemical pure grade, hydroxyl value = 175–185 mg KOH g^−1^) and dibutyltin dilaurate (DBTDL) were purchased from Sinopharm Chemical Reagent Co. Ltd., Shanghai, China. 2,2-Bis(hydroxymethyl)propionic acid (DMPA), isophorone diisocyanate (IPDI), triethylamine (TEA), and ethylenediamine (EDA) were purchased from Shanghai Aladdin Reagent Company (Shanghai, China) with the highest purity. Polypropylene glycol 2000 grade (PPG) was purchased from Guangzhou Hengyu Chemicals Company (Guangzhou, China). Phenolic epoxy resin (chemical pure grade, epoxy value = 0.44) was gifted by Guangzhou XiLu Chemical Co. Ltd., Guangzhou, China).

### 3.2. Construction of Micro-Buckling and Wrinkling Patterned Surface

The matt polyurethane (PU) coating was synthesized from an optimized waterborne procedure. Castor oil and phenolic epoxy resin were used as the modified agent, which increased the degree of branching and cross-linking density of PU, and also contributed to the mechanical instability during the film formation. First of all, 40 g of PPG and a certain amount of CO were added to a flask. Then, the flask was vacuumed and exchanged with high pure N_2_ atmosphere. Pre-polymerization was conducted at 90 °C by adding 17.58 g IPDI under catalyst of 0.01 g DBTDL for 2 h. Further, DMPA and phenolic epoxy resin (accounted for PPG weight seen in Table 1) were added to further react for 3 h. TEA (also accounted for PPG weight seen in Table 1) was added to neutralize the DMPA at 50 °C for 0.5 h. Lastly, the neutral pre-polymer was dropwise added into a new reactor filled with aqueous EDA solution at a stirring speed of 1000 r min^−1^. The addition of EDA contents in molar ratio was equal to the theoretical remaining NCO contents. It aimed to ensure the complete reaction of the remaining NCO groups, which were monitored by FTIR at 2270 cm^−1^ absorption band. The level of buckling and wrinkling could be adjusted by varying the CO contents from 1.72 wt% to 5.16 wt% of PPG weight (Seen in Table 1). However, the smooth surface of a PU coating could be achieved without addition of phenolic epoxy resin. The PU emulsions were poured onto a 10 cm × 10 cm Teflon plate and dried at ambient temperature for 24 h, and further vacuumed at 60 °C for 24 h. The dried films had a thickness of ~150 μm. They were stored in vacuum container for further analysis. In Table 1, E-44 is the abbreviation of phenolic epoxy resin with epoxy value = 0.44.

### 3.3. Characterization

Atomic Force Microscopy (AFM) measurements were carried out at ambient temperature by a Bruker Multimode 8-HR atomic force microscope with a maximum of 125 μm × 125 μm measurement length and a maximum of 10 μm vertical range. The micro-scope was placed on an active isolation table standing on a large iron table to eliminate external vibrations. All pictures were gained at ScanAsyst^®^ measurement mode with a pixel resolution of 512 × 512. The XY resolution is better than 0.1 nm and the Z resolution is 0.01 nm. The resulted pictures were further analyzed and processed with Nano Scope Analysis 1.8 software (Bruker Company, Santa Barbara, CA, USA).

Three-dimensional (3D) surface profilometer produced by BMT Company is an opto-electronic 3D measurement system for non-contact measurement and analysis for surfaces. The configuration includes a precise measurement stand with granite bridge, translation XY-stages, a confocal chromatic sensor, and a system controller. The lateral resolution is better than 1 μm and the vertical resolution is 1 nm. The acquisition of mean data is achieved using the proprietary BMT 3D software package. The images were obtained on two different lengths of measurement (2 mm and 5 mm) with an array of 751 × 751 measured heights. The data were corrected for sample tilt. Such corrections only make tilted surface at the same horizontal plane, which have no influence on the surface roughness and the specular gloss.

The topographies of film surfaces were obtained by a Hitachi S-3400N Scanning Electronic Microscope (SEM) at 25 °C, in a vacuum, and at a 15 kV accelerating voltage. Prior to the SEM analysis, the dried films were pasted on copper plate with conductive adhesive tape and were further sprayed with gold powder.

The glosses were tested with a micro-TRI-gloss GB4430 gloss meter (BYK-Gardner Columbia, MD, USA). A white and a black standard panel were simultaneously used for calibrating the standard gloss. The measured gloss is an average value of 10 measurements at angles of incident light of 20°, 60° and 85°. The angles for the gloss measurements were selected as defined in the DIN 67530, ISO 2813, and ASTM D-523 standards. The standard deviation of the gloss measurements was less than 2.0 gloss units.

The roughness parameters (*S_a_*, *S_q_*, *S_p_*, *S_v_*, *S_z_*, *S_ku_*, *S_sk_*, etc.) in this research are based on the complete 3D surface, which give more significant values. Calculations of these roughness parameters have been conducted using the built-in software of the corresponding instruments. *S_a_* refers to the arithmetical mean deviation of the roughness evaluated over the calculated 3D surface:

$$S_{a}=\underset{a}{\iint }\left|Z\left(x,y\right)\right|d\left(x\right)dy$$

*S_q_* refers to the root mean square deviation of the roughness evaluated over the calculated 3D surface:

$$S_{q}=\underset{a}{\iint }{\left(Z\left(x,y\right)\right)}^{2}d\left(x\right)dy$$

*S_p_* refers to the maximum peak height deviation of the roughness evaluated over the calculated 3D surface.

*S_v_* refers to the maximum valley depth deviation of the roughness evaluated over the calculated 3D surface.

*S_z_* refers to the maximum height deviation of the roughness evaluated over the calculated 3D surface, which can be expressed by

$$S_{z}=S_{p}-S_{v}$$

*S_ku_* refers to the Kurtosis of the 3D surface texture. A histogram of the heights of all measured points is established and the deviation from an ideal normal distribution is represented by *S_ku_*:

$$S_{ku}=\frac{1}{S_{q}^{4}}\underset{a}{\iint }{\left(Z\left(x,y\right)\right)}^{4}d\left(x\right)dy$$

*S_sk_* refers to the Skewness of the 3D surface texture. A histogram of the heights of all measured points is established and the deviation from an ideal Normal distribution is represented by *S_sk_*:

$$S_{sk}=\frac{1}{S_{q}^{3}}\underset{a}{\iint }{\left(Z\left(x,y\right)\right)}^{3}d\left(x\right)dy$$

## 4. Results and Discussion

### 4.1. Effect of CO Content on Surface Morphology

**Figure 2:**
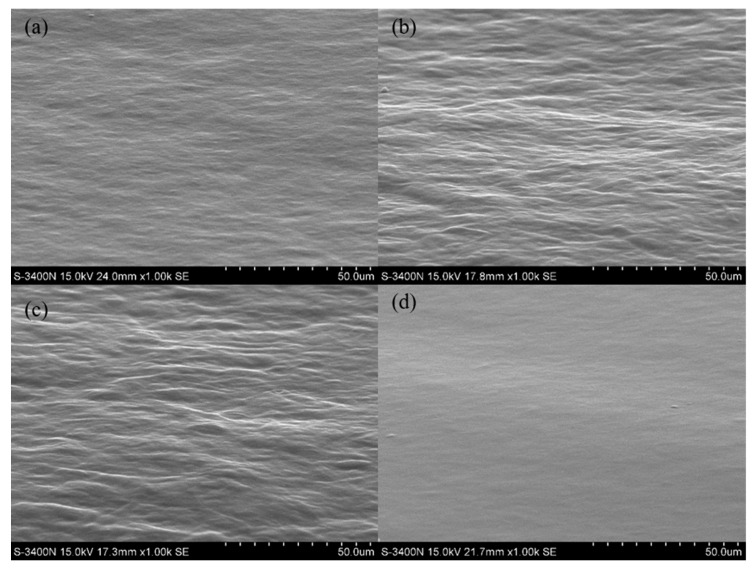
SEM topographic images of *PU1*, *PU2*, *PU3*, and *PU4* films, respectively. The CO contents are (**a**) 1.72%, (**b**) 3.44%, (**c**) 5.16%, and (**d**) 6.88%, without the addition of phenolic epoxy resin.

The SEM images were obtained for the tilted samples at 60° angle shown in Figure 2. Figure 2a,c shows the typical SEM topographic images for PU films with the CO contents of 1.72%, 3.44%, and 5.16% respectively. In contrast, Figure 2d demonstrates the surface morphology of PU film with the CO content of 6.88% but without addition of phenolic epoxy resin.

From the above images, it is greatly obvious that PU film surfaces of Figure 2a–c are becoming much rougher. There are many microscale wave-crests and wave-troughs shown on the surfaces and the uneven levels increase regularly with increasing CO contents from 1.72% to 5.16%. This result indicates that the level of branch and cross-linking of PU enhanced a lot due to the increase of CO contents. However, the PU film of Figure 2d presents a highly smooth and flat surface with the CO content up to 6.88%. The only composition difference between Figure 2a–c and Figure 2d is that there was no addition of phenolic epoxy resin in *PU4* product. This reveals that phenolic epoxy resin played an important role in constructing the buckling and wrinkling patterned surface of PU coating.

### 4.2. Formation Mechanism of Buckling and Wrinkling Patterned Surface

A reasonable hypothesis for the formation mechanism of buckling and wrinkling patterned surface is proposed. Due to the introduction of epoxy resin, parts of modified epoxy polyurethane and parts of unmodified polyurethane with epoxy showed the modulus mismatch [23,24,25] in the process of film formation. This modulus mismatch caused the formation of buckling and wrinkling patterned surface in a micro-scale. Because the phenolic epoxy resin has a large amount of active hydroxyl groups and epoxy groups, which was capable of reacting with the remaining isocyanate. Therefore, the partially epoxy modified polyurethane macromolecules had a larger molecular volume and lower specific gravity compared to the partially unmodified polyurethane. The modified epoxy polyurethane macromolecules floated on the surface more easily and brought together with surrounding molecules to form a buckle or a winkle band during the film formation. At the macroscopic level, it could be considered to the modulus mismatch between the partially modified epoxy polyurethane and partially unmodified polyurethane. In general, there are two conditions that need to be met to construct a buckling and wrinkling patterned surface of PU coating: (1) the PU coating should be cross-linked enough to have the swellability and elastic modulus, like addition of CO to promote cross-linking structure; and (2) there needs to be an additional phenolic epoxy resin to fabricate the modulus mismatch in PU system.

### 4.3. Effect of Measurement Length Scale on Roughness

**Figure 3:**
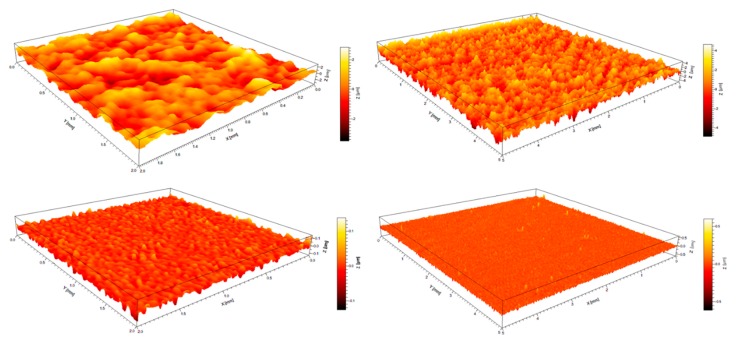
3D morphologies of typical *PU2* and *PU4* films in 2 mm (**left**) and 5 mm (**right**) length scales. Upper images are corresponding to *PU2* and lower images to *PU4* film.

The morphologies of typical *PU2* and *PU4* films in 2 mm (left) and 5 mm (right) length scales are shown in Figure 3. These photographs were captured by 3D surface profilometer and all kinds of area roughness parameters were calculated. The surface of *PU2* (upper) was rougher than that of *PU4* (lower); the concave and convex areas were distinctly obvious in *PU2*. It should also be observed that the scale values of the four-color bar increased with increasing the measurement length from 2 mm to 5 mm. This result shows that more wave-peaks and wave-troughs were detected due to the extent of measurement lengths. The area roughness parameters were listed in Table 2.

**Figure 4:**
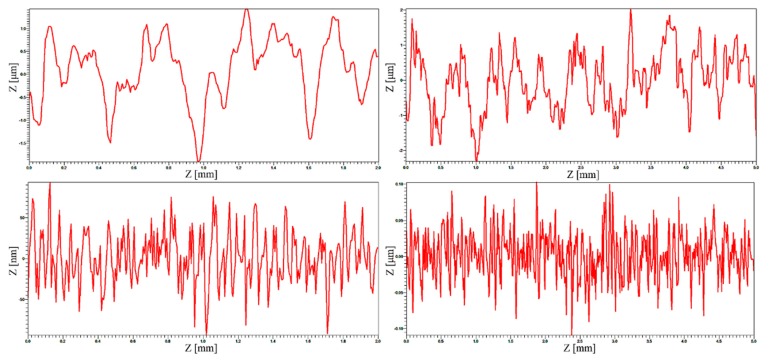
Surface profile curves of typical *PU2* and *PU4* films extracted from the above Figure 3. Upper images are corresponding to *PU2* and lower images to *PU4* film.

A better view of surface roughness can be seen in line scanning curves. Figure 4 shows the profile curves of *PU2* (upper) and *PU4* (lower) films extracted from Figure 4. In terms of *PU2* film, the highest peak and lowest peak were 1.42 μm and −1.90 μm, respectively, in measurement length of 2 mm, whereas in the 5 mm scale, the highest peak went upward to 2.02 μm and the lowest peak reached to −2.30 μm. With respect to *PU4* film, the highest peak and the lowest peak were 94 nm and −95 nm, respectively, in 2 mm length scale, whereas the highest peak increased a little to 103 nm and the lowest peak approached to 110 nm in 5 mm measurement length. These results reflect the changes of the following roughness values to some extent.

**Figure 5:**
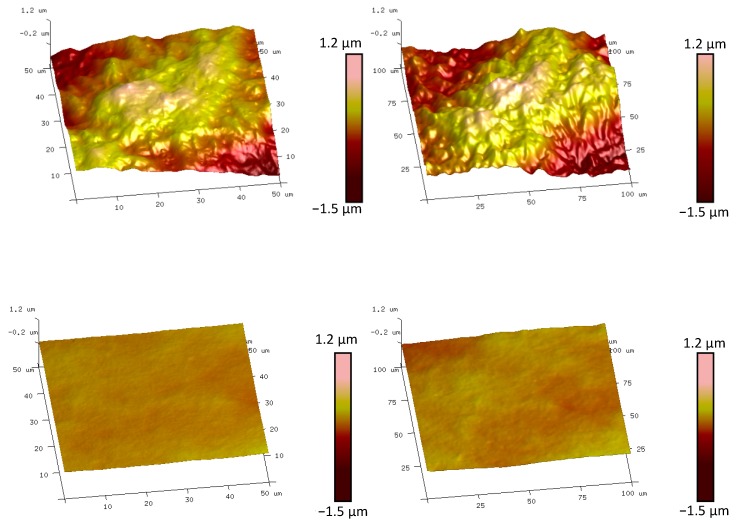
AFM ScanAsyst^®^ mode height images of *PU2* and *PU4* at 50 μm × 50 μm and 100 μm × 100 μm measurement lengths, respectively. Upper images are corresponding to *PU2* and lower images to *PU4* film.

AFM was also employed to investigate the 3D morphologies and calculate the roughness parameters in a smaller micro scale compared to 3D surface profilometer. Figure 5 demonstrates the AFM height images of *PU2* and *PU4* films at ScanAsyst^®^ measured mode. It is evident that *PU2* film surfaces at both 50 μm × 50 μm and 100 μm × 100 μm measurement lengths were more uneven than that of *PU4*. Therefore, the roughness degrees of *PU2* were much greater than that of *PU4*. Besides that, the two sets of images also show that the roughness degrees increased slightly when the measuring ranges were enlarged from 50 μm × 50 μm to 100 μm × 100 μm. To have a better comparison of the roughness parameters at a wide range of measurement length scale, we put the calculated roughness parameters from AFM and the calculated roughness parameters from 3D surface profilometer at the same table, that is, Table 2.

As shown in Table 2, we can see clearly that the arithmetic average roughness *S_a_* and the root mean square (RMS) roughness *S_q_* (most commonly used roughness parameters) of *PU2* increased remarkably from 0.23 μm to 0.68 μm and from 0.30 μm to 0.88 μm, respectively, with the increase of measurement length. Similarly, the *S_a_* and *S_q_* of *PU4* increased from 0.025 μm to 0.20 μm and from 0.033 μm to 0.26 μm, respectively, when the measurement scale extended from 50 μm × 50 μm to 5000 μm × 5000 μm. Other three roughness parameters including *S_p_*, *S_v_*, and *S_z_* also stepped up with increasing the length of measurement.

Kurtosis is a measure of whether the data are heavy-tailed or light-tailed relative to a normal distribution. The kurtosis of any univariate normal distribution is 3. It is common to compare the kurtosis of a distribution to this value. Distribution with kurtosis less than 3 produces fewer and less extreme outliers than does the normal distribution. Distribution with kurtosis greater than 3 has tails that asymptotically approach zero more slowly than a Gaussian, and therefore produces more outliers than the normal distribution. In other words, kurtosis is a descriptor of the shape of a probability distribution. From the *S_ku_* values of Table 2, *S_ku_* > 3 and *S_ku_* < 3 seemed to be random on the different measuring ranges. Actually, it depended on which sections accounted for more between raised sections and troughed sections. That is, it was highly related to the measuring areas that we chose. This result indicates that the concave and convex areas were distributed occasionally on the surface.

Skewness is used to describe asymmetry from the normal distribution in a set of statistical data. The skewness for a normal distribution is 0, and any symmetric data should have skewness near 0. Negative values for the skewness indicate data that are skewed left and positive values for the skewness indicate data that are skewed right. By skewed left, we mean that the left tail is long relative to the right tail. Similarly, skewed right means that the right tail is long relative to the left tail. It is interesting to find that the absolute value of *S_sk_* of *PU2* and *PU4* samples decreased a lot and approached zero with the growth of measuring length. This is due to that in the smaller scanning size the raised and troughed areas had a great effect on the asymmetry of the tested surface, whereas in contrast, these small convex and concave sections only exerted a limited influence on the deviation of distributed morphology in the larger scanning area.

**Figure 6:**
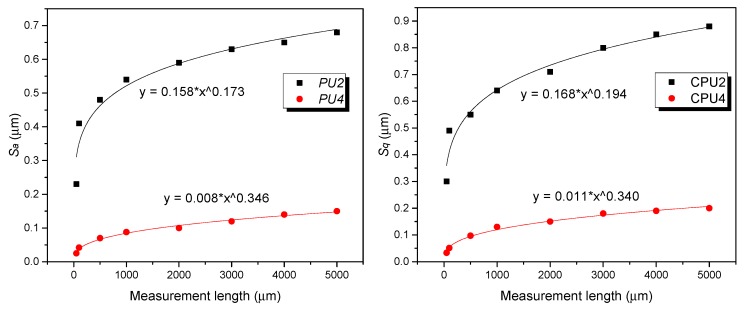
Surface roughness *S_a_* and *S_q_* as a function of measurement length for *PU2* and *PU4*, respectively.

To establish the functional relationship between the surface roughness and the measurement length scale, more surface roughness values are required at various length scales. Thus, we employed the built-in software of 3D surface profilometer to cut-off the large images into small ones. This method enables the calculation of roughness parameters on a wide range of measuring length quite easily. The 500 μm × 500 μm and 1000 μm × 1000 μm images were obtained from the 2000 μm × 2000 μm images. Similarly, the 3000 μm × 3000 μm and 4000 μm×4000 μm micrographs were cut-off from 5000 μm × 5000 μm images. The corresponding surface roughness *S_a_* and *S_q_* were calculated from these cut-off images. Figure 6 shows the variations of the surface roughness *S_a_* and *S_q_* with the measurement length, illustrating the length-scale dependence of roughness. The roughness initially increased rapidly and then slowly with increasing the length scale. What is more, a typical power function y = a*x^b for describing the relation between the surface roughness and the length scale was adapted to all samples. However, the *PU2* values of R^2^ of both images were up to 0.98, and the *PU4* of R^2^ of both images were less than 0.92. This result demonstrates that the correlation between roughness and measured length decreased with increasing the surface roughness.

### 4.4. The Gloss at Different Incident Angles

In Table 3, the gloss of all samples at three typical incident angles of 20°, 60°, and 85° were obtained. It is distinct that *PU4* had the highest gloss at three angles of incidence compared to other samples. The gloss values of *PU4* at 20°, 60°, and 85° were 66.8, 94.5, and 123.6, respectively. In *PU1–PU3* samples, the glosses at varied incident angles decreased with the increase of CO contents. The gloss units of *PU1–PU3* at 20°, 60°, and 85° decreased from 21.4, 53.6, and 90.4 to 8.0, 19.0, and 51.4, respectively. What is more, the gloss showed a reverse trend relative to the surface roughness. As the surface roughness of a film increased, the gloss decreased instead, and thus the film had a dull or lusterless surface.

A further comparison of matting effects at three typical angles of viewing is often necessary as matting effect varies with the incident angle. Due to the glossing up effect, the gloss values at 85° are invariably larger than that of at 60° and 20° [26,27]. However, in Table 3, the great differences of gloss values at 20°, 60°, and 85° incident angles demonstrated that the surface roughness of films was in a minimal microscale, which was hard to be seen in naked eyes. The lower variability of gloss values at different incident angles was often related to the larger uneven surface, which was mostly caused by injection of fillers or pigments. Therefore, through the investigation of gloss at different incident angles, we can conclude that the synthesized samples could show an extremely delicate and micro-rough surface compared to the conventional matt coatings by addition of matting agent.

### 4.5. Effect of Surface Roughness on Gloss

**Figure 7:**
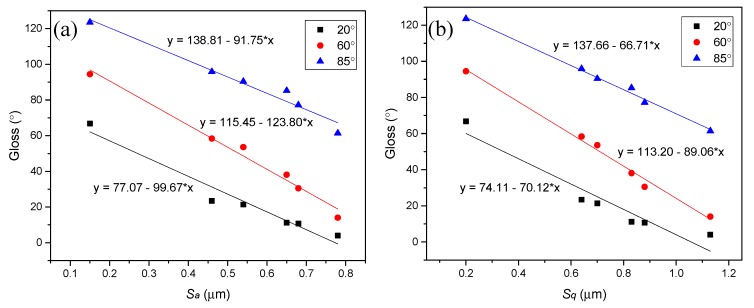
Gloss as a function of surface roughness (**a**) *S_a_* and (**b**) *S_q_* calculated from 5000 μm × 5000 μm images.

Figure 7 shows the relationship between the gloss and the surface roughness for randomly distributed micro rough surfaces. The *S_a_* and *S_q_* values for each sample were calculated from 5000 μm × 5000 μm images. The relationship could be expressed with linear fit function y = a + b*x. The R^2^ values of Figure 7a were 0.95, 0.97, and 0.98 at 20°, 60° and 85° incident angles respectively. Similarly, the R^2^ values Figure 7b were 0.90, 0.98, and 0.99 at 20°, 60° and 85° incident angles respectively. The strong correlations (R^2^ > 0.90) indicated the great dependence of the gloss on the surface roughness. It is clear that the gloss at three typical angles of incidence decreased linearly with the increasing of the surface roughness *S_a_* and *S_q_*. In addition, the R^2^ values increased from 0.95 to 0.98 for S*_a_* and from 0.90 to 0.99 for S*_q_* when increasing the light incident angles from 20° to 85°. This concludes that the gloss-meter of 85° incident angle was more accurate to determine the gloss of matt PU coating.

## 5. Conclusions

In this study, the surfaces of matt polyurethane coating were well-analyzed by SEM, AFM, and 3D surface profilometer, and the correlations of surface roughness on measurement length and gloss were well-established. The SEM demonstrates that there were many microscale wave-crests and wave-troughs shown on the surface and the uneven level increased regularly with increasing CO contents from 1.72% to 5.16%. The AFM and 3D surface profilometer were used to analyze surface morphologies and to calculate a series of surface roughness parameters at a wide range of measured lengths. The typical power function y = a*x^b was adapted for describing the relation between the surface roughness and the length scale, and the correlation between roughness and measured length decreased with increasing the roughness. The linear fit function y = a + b*x could be used to illustrate the correlation between the gloss and the surface roughness, and the gloss-meter of 85° incident angle is the best to describe the gloss of matt PU coating.

## Figures and Tables

**Table 1:** Designations of wrinkling patterned waterborne cross-linked PU.

Designation	DMPA (wt %)	CO (wt %)	E-44 (wt %)	TEA (wt %)
***PU1***	9.59	1.72	9.23	7.23
***PU2***	9.59	3.44	9.23	7.23
***PU3***	9.59	5.16	9.23	7.23
***PU4***	9.59	6.88	0	7.23

**Table 2:** Surface roughness parameters calculated from atomic force microscopy (AFM) and 3D surface profilometer.

Designation	Measurement Length (μm)	*S_a_* (μm)	*S_q_* (μm)	*S_p_* (μm)	*S_v_* (μm)	*S_z_* (μm)	*S_ku_*	*S_sk_*
*PU2*	50 × 50	0.23	0.30	0.74	−1.03	1.77	3.25	−0.50
	100 × 100	0.41	0.49	1.12	−1.36	2.48	2.40	−0.31
	2000 × 2000	0.59	0.70	1.96	−2.46	4.42	2.28	−0.12
	5000 × 5000	0.68	0.88	4.08	−3.70	7.78	3.39	−0.06
*PU4*	50 × 50	0.025	0.033	0.13	−0.11	0.24	4.75	0.46
	100 × 100	0.042	0.051	0.17	−0.18	0.35	2.59	−0.31
	2000 × 2000	0.1	0.16	0.23	−0.22	0.45	3.78	−0.15
	5000 × 5000	0.15	0.20	0.52	−0.45	0.97	2.74	−0.07

**Table 3:** The gloss values of wrinkled waterborne cross-linked PU at different angles of incidence.

Designation		Gloss	
	20°	60°	85°
*PU1*	21.4	53.6	90.4
*PU2*	10.7	30.5	77.2
*PU3*	8.0	19.0	51.4
*PU4*	66.8	94.5	123.6
